# Sex Differences in Frail Older Adults with Foot Pain in a Spanish Population: An Observational Study

**DOI:** 10.3390/ijerph17176141

**Published:** 2020-08-24

**Authors:** Emmanuel Navarro-Flores, Carlos Romero-Morales, Ricardo Becerro de Bengoa-Vallejo, David Rodríguez-Sanz, Patricia Palomo-López, Daniel López-López, Marta Elena Losa-Iglesias, César Calvo-Lobo

**Affiliations:** 1 Frailty Research Organizaded Group (FROG), Department of Nursing, Faculty of Nursing and Podiatry, University of Valencia; 46010 Valencia, Spain, emmanuel.navarro@uv.es; 2 Faculty of Sport Sciences, Universidad Europea de Madrid, 28670 Madrid, Spain; 3 Facultad de Enfermería, Fisioterapia y Podología, Universidad Complutense de Madrid, 28040 Madrid, Spain; ribebeva@ucm.es (R.B.d.B.-V.); davidrodriguezsanz@ucm.es (D.R.-S.); cescalvo@ucm.es (C.C.-L.); 4 University Center of Plasencia, University of Extremadura, 06006 Badajoz, Spain; patibiom@unex.es; 5 Research, Health and Podiatry Group, Department of Health Sciences, Faculty of Nursing and Podiatry, Universidade da Coruña, 15403 Ferrol, Spain; daniellopez@udc.es; 6 Faculty of Health Sciences, Universidad Rey Juan Carlos, 28933 Madrid, Spain; marta.losa@urjc.es

**Keywords:** frailty, older adults, foot deformities, foot diseases, foot pain

## Abstract

Frailty is a condition that can increase the risk of falls. In addition, foot pain can influence older adults and affect their frail condition. The main objective was to measure the frailty degree in older adults in a Spanish population with foot pain from moderate to severe. Method: This is a cross-sectional descriptive study. A sample of people older than 60 years (*n* = 52), including 26 males and 26 females, were recruited, and frailty disability was measured using the 5-Frailty scale and the Edmonton Frailty scale (EFS). Results: Spearman’s correlation coefficients were categorized as weak (rs ≤ 0.40), moderate (0.41 ≤ rs ≥ 0.69), or strong (0.70 ≤ rs ≥ 1.00). There was a statistically significant correlation for the total score (*p* < 0.001) and most of the subscales of the 5-Frailty scale compared with the EFS, except for Mood (*p* > 0.05). In addition, females and males showed similar 5-Frailty and Edmonton Frail scales scores with no difference (*p* > 0.05). Conclusion: Foot pain above 5 points, i.e., from moderate to severe, does not affect the fragility more in one sex than another.

## 1. Introduction

Aging and chronical illness processes, like hyperglycemic disease, as well as muscle skeletal and heart processes can produce frailty syndrome, and as a consequence of this, be degenerative and exhibit some alterations that can affect one’s mental and general health [1]. For example, aging and frailty can affect gait speed and increase fall risk due to balance alterations [2,3,4]. Furthermore, the presence of frailty symptoms affects the health-related quality of life (HQoL) [5] in this population group.

We can define frailty syndrome as a group of health alterations that can affect several aspects, such as the psychological, biological and social aspects, as it is a consequence of a dynamic process that reduces a person’s health status [6]. Regarding foot conditions in older adult populations, foot disorders and diseases are present most frequently in the frailty population group, comprising approximately 25% [7,8].

The frequency of a frail state in people older than 65 years has been estimated between 4 and 59.1% [9].

Consultations at general practitioners related to ankle and foot conditions of osteoarticular pain origin account for more than 8% [10]. Accordingly, suffering may raise this predominance in older adults who have characteristic foot requirements that can be akin to bigger disorders [11], foot health-related quality of life (HQoL) [12], and risk of falls [13,14]

The 5-Frailty scale is a questionnaire of 5 items, which was established using an auto-administered dimension [6]. Respondents can provide affirmative or negative answers, with one punctuation to the positive response. For measuring the degree of frailty, respondents can score between zero and five points, and subjects are qualified as robust (zero point), pre-frail (one to two points), or frail (three points). The level represents their respective tiredness, resistance, ambulation, disease, and loss of weight.

Tiredness is evaluated by asking subjects if they felt tired; resistance was determined by each subject’s report on their capacity to climb stairs; ambulation is represented by each subject’s information on their ability to move around; illness is determined by the presence of more than five of a total of eleven pathologies, including cardiovascular diseases, diabetes, and loss of weight by a reduction of five percent during the last year [15].

The Edmonton Frailty scale (EFS) assesses nine subscales: (1) cognitive, (2) general health status, (3) independence, (4) social support, (5) pharmacologic treatment, (6) feeding, (7) mood, (8) continence, and (9) functional performance, using eleven questions. The maximum score is 17 and represents the highest degree of frailty [16]. In this case, a frailty score between zero and four does not present frailty, scores of five to six represent apparently vulnerable, scores of seven to eight represent fair frailty, scores of nine to ten represent frailty moderate, and scores of eleven or more represent severe frailty [17].

No study has yet correlated the scores of the EFS and the 5-Frailty scale. Therefore, the goal of this research was to correlate the subscales of the EFS and 5-Frailty scale in older adults and those with related foot pain.

In the literature, no references have been found regarding the frailty of older adults with foot pain related to sex, and therefore our hypothesis is that there are differences in the levels of frailty in older adults with foot pain related to sex. The objective of this study is to determine if sex can influence a greater degree of frailty.

## 2. Materials and Methods

The study was developed in Spain. We recruited older adult patients in a medical center, a rehabilitation service, and podiatry clinics in the Generalitat of Valencia, and all survey data were collected between September 2019 and January 2020. Researchers obtained signed informed consent from all subjects, and observational research was carried out using STROBE [18].

Considering sex distribution as the independent variable to calculate the sample size, G* Power 3.1.9.2 software (Heinrich-Heine-Universität Düsseldorf; Düsseldorf, Germany) was used after considering testing the sex differences between two independent means about sex, comparing their frailty scores. A two-tailed hypothesis, a large effect size of 0.8, an error of α = 0.05 with a 95% confidence interval, an error β = 20%, and a power analysis of 1 − β = 0.80 were considered. Consequently, a total sample size of 52 subjects with 26 in each group was included in this study.

Prior to beginning the research, approval for conducting this study was obtained from the Ethics Committee of the University of Extremadura, 1/2020.

Informed consent was obtained from each participant after the purpose and process of the study were explained and the privacy of the participants’ information was assured. The fact that their participation was entirely voluntary was also highlighted.

The inclusion criteria comprised adult patients older than 60 years who presented foot pain during the last 6 months due to toe or foot deformities, regardless of its origin or cause; higher than five points in the VAS score, excluding wounds; and able to communicate orally and provide written informed consent. VAS scores above five points, i.e., from moderate to severe, showed an intraclass correlation coefficient reliability of 0.870 [19].

The exclusion criteria were major neurocognitive disorder; patients who did not answer initial identification questions or who did not understand the rules of participation; and those who refused to participate in the research, i.e., no signed consent.

To recruit volunteer participants, we posted recruitment flyers in senior centers’ gathering places. We also addressed groups of older adults in the center to invite them to contact us if they were willing to participate in the study. Once a potential participant expressed interest, a cognitive function evaluation was performed by a gerontological nurse practitioner (GNP) to establish the cognitive eligibility of the participant. Following evaluation by the GNP, the investigators explained the study procedures in detail to the participants.

The interview was composed of general questions of general health status, socio demographic characteristics (sex, age, BMI, height, and weight), comorbidities (e.g., anxiety, depression, diabetes, obesity, osteoarticular diseases, vascular disorders, and kidney illness) collected from medical records. Furthermore, specific items related to foot pain, such as the actual treatment or foot deformities, were assessed by a senior podiatry physician (ENF).

In this study, a total of 65 older adults expressed interest in study participation, and all met the cognitive requirements. The participants all attempted to complete the survey questionnaires. Ultimately all surveys were analyzed for the study; 14 surveys were excluded due to incomplete answers. For participants who were not able to read the questionnaires due to vision problems, the investigators read the questions aloud and marked the participants’ answers on the questionnaires. Participants took about 15 min to complete the questionnaires. Participants did not get any compensation for their participation in the study.

### 2.1. Evaluation of Frailty

The EFS was designed to measure frailty in nine subscales: cognitive, general health status, independence, social support, pharmacologic treatment, feeding, mood, continence, and functional performance [16,20]. Total scores range from 0 to 17, and higher scores indicate more frailty, ranging from 1 = not frail; 2 = ostensibly susceptible frail; 3 = almost never; to 4 = almost always. The EFS ranged by 3 degrees. The Cronbach’s α for the EFS was 0.93 [21].

First, a strong frailty mark and subjects without frailty were classified using the EFS. Subjects who scored less than five points were designated as not frail. Secondly, ostensibly susceptible frail subjects were designated as those who obtained six to eleven points. The third group included those who scored between twelve and seventeen points. The questionnaire administration required only 15 min to complete.

Participants also completed the 5-item Frailty scale [22], which has been previously validated into Spanish with an ICC = 0.82 [6,23]. This scale measured five subscales: tiredness, resistance, go around, disease, and loss of weight. Frailty scores ranged from 0 to 5, and higher scores indicate more frailty. Those participants that scored between three and five were considered frail, those that scored one or two were considered to be pre-frail, and those that obtained zero points were considered to be not frail.

The authors obtained permission from the original authors of the EFS and 5-Frailty scale to use their clinimetric tool to measure the frailty degree.

### 2.2. Statistical Analysis

All the variables were normally distributed, as determined by the Kolmogorov–Smirnov test (*p* > 0.05).

Regarding the results of the quantitative variables, the non-parametric data were described in terms of their median, interquartile range (IR), and minimum and maximum (range) values. Parametric data were described using means, the standard deviation (SD), and minimum and maximum (range) values.

A comparison of the quantitative data between males and females for the different questionnaire subscales of the EFS and 5-Frail scale was conducted, and significant differences were checked using an independent Student’s *t*-test. Non-normal data were analyzed using Mann–Whitney U tests.

Spearman’s correlation coefficients (rs) were determined between tests and qualified as low rs ≤ 0.40, moderate 0.41 ≤ rs ≥ 0.69, or solid 0.70 ≤ rs ≥1.00.

All analyses were considered statistically significant when the *p*-value < 0.05 with a 95% confidence interval (CI). Statistical analyses were made in SPSS (V.26.0, Chicago, IL, USA).

## 3. Results

### 3.1. Descriptive Data and Socio-Demographic Data

A normal distribution for age, height, weight, and BMI was shown (*p* > 0.05), and all items from the 5-Frailty test and the EFS showed no normal distribution (*p* < 0.05).

The size sample included 52 subjects whose mean age was 77.47 ± 10.69 years. The study subjects included 26 (50.00%) females and 26 (50.00%) males. Table 1 shows the socio-demographic characteristics. Males and females did not show statistically significant socio-demographic differences (*p* > 0.05) for age or BMI, although a higher weight and height (*p* < 0.05) were shown for males compared with females. There was no difference in the intensity of pain between sexes (*p* = 0.561).

### 3.2. Edmonton Frail Scale and 5-Frailty Scale Sex Distribution

As shown in Table 2, the 5-Frailty scale scores did not manifest any statistically significant difference (*p* > 0.05) for the subscales nor for the total scores between females and males with foot pain. Furthermore, the EFS scores by sex distribution are shown in Table 3, whose subscales did not show statistically significant differences (*p* > 0.05). The results of both test show that frailty in males and females with foot pain is similar.

Table 4 shows a good correlation between items of both tests, except for the subscale nutrition (*p* = 0.490).

## 4. Discussion

This investigation aimed to compare the frailty degree differences between males and females with foot pain from moderate to severe using the 5-Frailty Scale and EFS. Indeed, frailty did not show statistically significant differences between the sexes. Although the total frailty score Spearman correlation was significant in Table 4, it is worthy to note that the correlation was modest, even when comparing domains, which were expected to correlate with functional independence and performance from the EFS and with resistance and ambulation from the 5-Frailty scale.

Both scales are correlated, which confers concurrent validity of each subscale to recent studies and sustains the application of the 5-Frailty score as an acceptable measurement related to frailty aspects such as ambulation, illness, or loss of weight. This aspect can be considered as an advantage with respect to other frailty scales adapted to Spanish to evaluate specific frailty aspects, like the Frailty Trait Scale (FTS) [24].

Moreover, the results from this study are different from those obtained by other researchers in functional performance related to foot pain [25,26]. Our results did not show statistically significant differences related to foot pain [5,8]. These findings are different, with similar research reported on subjects with foot pain [1], as well as with foot pain [27] as measured by employing the visual analogic scale (VAS) to determine the sex differences in healthy subjects with foot problems.

There was a statistically significant correlation in the total score and most of the subscales of the 5-Frailty scale compared with the EFS, except for Mood (*p* > 0.05).

Furthermore, prior studies have identified females who suffered from fibromyalgia and had developed foot problems as a consequence had an increased frailty degree [28].

Future studies should incorporate all other foot risk factors related with frailty syndrome. Despite the fact that the EFS has determined the frailty score [29,30].

Several limitations of this research should be taken into account. A population from different territories may be useful to ameliorate the strength of this research.

This research has only determined if foot pain can influence a greater degree of frailty by sex and we found that foot pain does not affect the frailty by sex.

Although ambulation and functional performance and risk of fall are very common in frail people [2,4], this research should also be developed for other population groups to determine the degree of frailty, for example, in widows who usually have higher frailty scores due to psychosocial aspects [29,31,32].

Furthermore, selective sampling can cause bias; for this reason, randomized sampling should be considered in future studies.

Ultimately, the correlation impact between the different foot disorders, including several genetics and acquired or traumatic alterations and chronic illness, was not studied in our research because the studied population was not suitably adjusted to develop these comparisons. The researchers therefore suggest that future research should be conducted considering different foot pathologies.

## 5. Conclusions

Foot pain above 5 points, i.e., from moderate to severe, does not affect the fragility more in one sex than another. Further research is needed considering different foot pathologies.

## Figures and Tables

**Table 1:** Descriptive and socio-demographic data of the sample.

Demographic and Descriptive Data	Total Group *n* = 52 Mean ± SD (Range)	Female *n* = 26 Mean ± SD (Range)	Male *n* = 26 Mean ± SD (Range)	*p* Value
Age (Years)	77.47 ± 10.69 (74.54–80.40)	79.07 ± 10.74 (75.16–82.98)	75.36 ± 10.50 (70.98–79.75)	0.224
Weight (kg)	62.47 ± 12.08 (59.16–65.78)	58.31 ± 12.44 (53.78–62.84)	67.95 ± 9.25 (64.09–71.82)	0.004
Height (m)	1.61 ± 0.08 (74.54–80.40)	1.57 ± 0.07 (1.54–1.59)	1.65 ± 0.07 (1.62–1.68)	0.000
BMI (Kg/m^2^)	24.19 ± 3.96 (23.10–25.27)	23.67 ± 4.30 (22.10–25.24)	24.87 ± 3.42 (23.45–26.30)	0.286
Foot Pain	7.18 ± 3.38 (23.10–25.27)	7.28 ± 1.36 (5.00–10.00)	7.05 ± 1.43 (5.00–10.00)	0.561

BMI: body mass index; Mean-standard deviation, range (min–max), and Student’s *t*-test for independent samples were applied. In all the analyses, *p* < 0.05 (with a 95% confidence interval) was considered statistically significant.

**Table 2:** Comparisons of the 5-Frailty scale scores between males and females.

Frailty Scale Domains	Total Group *n* = 52 Mean ± SD (Range) Median (IR)	Female *n* =26 Mean ± SD (Range) Median (IR)	Male *n* = 26 Mean ± SD (Range) Median (IR)	*p* Value
Fatigue	0.54 ± 0.50 (0.41–0.68) 1.00 (1.00)	0.55 ± 0.35 (0.35–0.74) 1.00 (1.00)	0.54 ± 0.50 (0.31–0.77) 1.00 (1.00)	0.965
Resistance	0.47 ± 0.50 (0.33–0.60) 0.00 (1.00)	0.51 ± 0.50 (0.32–0.71) 1.00 (1.00)	0.40 ± 0.50 (0.18–0.63) 0.00 (1.00)	0.448
Ambulation	0.47 ± 0.50 (0.30–0.59) 0.00 (1.00)	0.44 ± 0.50 (0.25–0.64) 0.00 (1.00)	0.45 ± 0.50 (0.22–0.68 0.00 (1.00)	0.965
Illness	0.43 ± 0.50 (0.29–0.57) 0.00 (1.00)	0.44 ± 0.50 (0.25–0.64) 0.00 (1.00)	0.40 ± 0.50 (0.18–0.63) 0.00 (1.00)	0.782
Loss of weight	0.54 ± 0.50 (0.41–0.69) 1.00 (1.00)	0.51 ± 0.50 (0.32–0.71) 1.00 (1.00)	0.59 ± 0.50 (0.36–0.81) 1.00 (1.00)	0.604
TOTAL Frailty Scale	2.49 ± 1.50 (2.08–2.91) 3.00 (3.00)	2.51 ± 1.45 (1.96–3.07) 3.00 (2.00)	2.45 ± 1.59 (1.74–3.16) 2.50 (3.00)	0.892

CI, Confidence Interval; IR: interquartile range. Mann–Whitney U tests were used. In all the analyses, *p* < 0.05 (with a 95% confidence interval) was considered statistically significant.

**Table 3:** Comparisons of the Edmonton Frail Scale scores between males and females.

Edmonton Frail Scale Domains	Total Group *n* = 52 Mean ± SD (95%CI) Median (IR)	Female *n* = 26 Mean ± SD (95%CI) Median (IR)	Male *n* = 26 Mean ± SD (95%CI) Median (IR)	*p* Value
Cognition	0.78 ± 0.64 (0.60–0.96) 1.00 (1.00)	0.79 ± 0.61 (0.55–1.02) 1.00 (1.00)	0.77 ± 0.68 (0.46–1.07) 1.00 (1.00)	0.865
General health status 2A	0.66 ± 0.52 (0.49–0.84) 1.00 (1.00)	0.65 ± 0.61 (0.42–0.88) 1.00 (1.00)	0.68 ± 0.64 (0.39–0.96) 1.00 (1.00)	0.907
General health status 2B	0.82 ± 0.88 (0.57–1.97) 1.00 (1.00)	0.79 ± 0.86 (0.46–1.12) 1.00 (1.00)	0.86 ± 0.94 (0.44–1.28 1.00 (1.00)	0.845
Functional independence	0.60 ± 0.85 (0.36–0.84) 1.00 (1.00)	0.58 ± 0.82 (0.27–0.89) 0.00 (1.00)	0.53 ± 0.90 (0.23–1.03) 0.00 (1.00)	0.923
Social support	0.49 ± 0.57 (0.32–0.65) 0.00 (1.00)	0.51 ± 0.57 (0.29–0.73) 0.00 (1.00)	0.45 ± 0.59 (0.19–0.71) 0.00 (1.00)	0.648
Medication use 5A	0.60 ± 0.49 (0.46–0.74) 1.00 (1.00)	0.58 ± 0.50 (0.39–0.77) 1.00 (1.00)	0.53 ± 0.49 (0.41–0.85 1.00 (1.00)	0.719
Medication use 5 B	0.54 ± 0.50 (0.41–0.69) 1.00 (1.00)	0.58 ± 0.50 (0.32–0.71) 1.00 (1.00)	0.50 ± 0.51 (0.27–0.72) 0.50 (1.00)	0.544
Nutrition	0.66 ± 0.47 (0.53–0.80) 1.00 (1.00)	0.65 ± 0.49 (0.47–0.83) 1.00 (1.00)	0.68 ± 0.47 (0.47–0.89) 1.00 (1.00)	0.843
Mood	0.54 ± 0.50 (0.4–0.69) 1.00 (1.00)	0.62 ± 0.49 (0.43–0.80) 1.00 (1.00)	0.45 ± 0.50 (0.22–0.68) 0.00 (1.00)	0.242
Continence	0.39 ± 0.49 (0.25–0.53) 0.00 (1.00)	0.44 ± 0.50 (0.25–0.64) 0.00 (1.00)	0.31 ± 0.47 (0.10–0.52) 0.00 (1.00)	0.351
Functional performance	1.05 ± 0.64 (0.87–1.24) 1.00 (0.00)	1.13 ± 0.58 (0.91–1.35) 1.00 (0.50	0.95 ± 0.72 (0.53–1.27) 1.00 (1.25)	0.332
Total Edmonton Frail Scale	6.969 ± 4.57 (5.67–8.24) 6.00 (7.00)	7.27 ± 4.30 (5.63–8.91) 7.00 (6.00)	6.54 ± 4.97 (4.34–8.75) 5.50 (6.50)	0.457

CI, Confidence Interval; IR: interquartile range. Mann–Whitney U tests were used. In all the analyses, *p* < 0.05 (with a 95% confidence interval) was considered statistically significant.

**Table 4:** Spearman’s correlations between the 5-Frailty scale and Edmonton Frail Scale score domains and totals.

Edmonton Frail Scale Domains	Fatigue r (*p*)	Resistance r (*p*)	Ambulation r (*p*)	Illness r (*p*)	Loss of Weight r (*p*)	TOTAL Frailty Scale r (*p*)
Cognition	0.428 (<0.001)	0.369 (0.002)	0.513 (<0.001)	0.386 (00.001)	0.498 (<0.001)	0.773 (<0.001)
General health status 2A	0.232 (0.059)	0.174 (0.160)	0.608 (<0.001)	0.576 (<0.001)	0.623 (<0.001)	0.750 (<0.001)
General health status 2B	0.269 (0.028)	0.194 (0.116)	0.641 (<0.001)	0.562 (<0.001)	0.624 (<0.001)	0.780 (<0.001)
Functional independence	0.295 (0.015)	0.131 (0.290)	0.497 (<0.001)	0.276 (0.024)	0.383 (00.001)	0.481 (<0.001)
Social support	0.259 (0.034)	0.107 (0.390)	0.521 (<0.001)	0.334 (0.006)	0.409 (0.001)	0.510 (<0.001)
Medication use 5A	0.401 (0.401)	0.115 (0.352)	0.627 (00.401)	0.006 (<0.001)	0.417 (<0.001)	0.683 (<0.001)
Medication use 5 B	0.069 (0.579)	0.152 (0.220)	0.579 (00.579)	0.621 (<0.001)	0.379 (0.002)	0.620 (<0.001)
Nutrition	−0.097 (0.438)	–0.080 (0.518)	0.282 (0.021)	0.326 (0.007)	0.299 (0.014)	0.324 (<0.001)
Mood	0.066 (0.594)	0.075 (0.548)	0.111 (0.369)	−0.158 (0.201)	0.067 (0.590)	0.066 (0.490)
Continence	0.078 (0.529)	0.302 (0.013)	0.571 (<0.001)	0.452 (<0.001)	0.469 (<0.001)	0.575 (<0.001)
Functional performance	0.492 (<0.001)	0.434 (<00.001)	0.076 (0.542)	−0.136 (0.271)	0.149 (0.228)	0.446 (<0.001)
Total Edmonton Frail Scale	0.329 (0.006)	0.276 (0.024)	0.624 (<0.001)	0.443 (<0.001)	0.582 (<0.001)	0.842 (<0.001)

Spearman correlation coefficients (r) and *p*-values were applied. In all the analyses, *p* < 0.05 (with a 95% confidence interval) was considered statistically significant.
